# Determination of Ammonium Ion Using a Reagentless Amperometric Biosensor Based on Immobilized Alanine Dehydrogenase

**DOI:** 10.3390/s111009344

**Published:** 2011-09-29

**Authors:** Ling Ling Tan, Ahmad Musa, Yook Heng Lee

**Affiliations:** 1 School of Chemical Sciences and Food Technology, Faculty of Science and Technology, Universiti Kebangsaan Malaysia, Bangi, Selangor 43600, Malaysia; E-Mail: babybabeoo@gmail.com; 2 Faculty of Science and Technology, Universiti Sains Islam Malaysia, Bandar Baru Nilai, Nilai, Negeri Sembilan D.K. 71800, Malaysia; E-Mail: andong@usim.edu.my

**Keywords:** biosensor, ammonium ion, reduced nicotinamide adenine dinucleotide, poly(2-hydroxyethyl methacrylate), alanine dehydrogenase

## Abstract

The use of the enzyme alanine dehydrogenase (AlaDH) for the determination of ammonium ion (NH_4_^+^) usually requires the addition of pyruvate substrate and reduced nicotinamide adenine dinucleotide (NADH) simultaneously to effect the reaction. This addition of reagents is inconvenient when an enzyme biosensor based on AlaDH is used. To resolve the problem, a novel reagentless amperometric biosensor using a stacked methacrylic membrane system coated onto a screen-printed carbon paste electrode (SPE) for NH_4_^+^ ion determination is described. A mixture of pyruvate and NADH was immobilized in low molecular weight poly(2-hydroxyethyl methacrylate) (pHEMA) membrane, which was then deposited over a photocured pHEMA membrane (photoHEMA) containing alanine dehydrogenase (AlaDH) enzyme. Due to the enzymatic reaction of AlaDH and the pyruvate substrate, NH_4_^+^ was consumed in the process and thus the signal from the electrocatalytic oxidation of NADH at an applied potential of +0.55 V was proportional to the NH_4_^+^ ion concentration under optimal conditions. The stacked methacrylate membranes responded rapidly and linearly to changes in NH_4_^+^ ion concentrations between 10–100 mM, with a detection limit of 0.18 mM NH_4_^+^ ion. The reproducibility of the amperometrical NH_4_^+^ biosensor yielded low relative standard deviations between 1.4–4.9%. The stacked membrane biosensor has been successfully applied to the determination of NH_4_^+^ ion in spiked river water samples without pretreatment. A good correlation was found between the analytical results for NH_4_^+^ obtained from the biosensor and the Nessler spectrophotometric method.

## 1. Introduction

The accurate measurement of NH_4_^+^ ion in an aquatic environment is of significant interest in environmental biological studies and the environmental evaluation of water since it is known to be toxic for aquatic organisms. In this sense, several methods have been proposed, most of them involving ion chromatography [1–3], potentiometry [4,5] or flow injection systems [6–11].

Most of the ion chromatography procedures proposed for its determination involve elaborate pre-column derivatization in some kind of matrix. Mori *et al.* [12] described a sensitive and rapid ion chromatography method to determine NH_4_^+^ ion in river waters. However, baseline separations of NH_4_^+^ from alkali and alkaline earth metal ions in water samples were non achievable. For potentiometric detection of NH_4_^+^ ion, nonactin has been widely used as sensing material. Even though nonactin-based ion-selective electrodes show good sensitivity toward NH_4_^+^ ion, they suffer interference from other ions such as K^+^ [13,14]. Flow injection systems combined with spectrophotometric methods, e.g., the Berthelot reaction involving a colour change in the presence of NH_4_^+^ ion, have very slow reaction kinetics [15], whereas fluorimetric flow injection analysis requires pretreatment of the samples with long diffusion times to avoid background interferences [2,16].

Today, there is a well-recognised trend towards the simplification and miniaturisation of analytical processes [2]. An amperometry approach employing a miniaturised SPE with immobilized enzyme as tranducer considerably improves the operation cost, providing for a simple, reliable, rapid and reproducible analytical procedure. A few biosensors for the amperometric determination of NH_4_^+^ ion employing glutamate dehydrogenase (GLDH) have been reported where the enzyme was immobilized onto the working electrode in several ways [17–19]. However, to effect the enzymic GLDH reaction, a substrate and co-factor normally needed to be introduced and this leads to an extra step during the assay of NH_4_^+^ ion. In order to obviate the needs for external reagent treatment during measurement, which may also cause contamination of the reference electrode, we describe in this work an approach employing a stacked membranes system for the immobilization of enzyme, co-factor and also substrate that eventually leads to a reagentless biosensor for NH_4_^+^ ion determination.

In this work, we have used alanine dehydrogenase (AlaDH) to construct a biosensor for the determination of NH_4_^+^ ion. To our knowledge, the use of AlaDH in an NH_4_^+^ ion biosensor has not been reported. The concept of the biosensor based on AlaDH is the reversible amination of pyruvate to L-alanine by AlaDH in the presence of NADH co-factor and NH_4_^+^ ion (Equation (1)) [20–22]. The current generated from the electrochemical process was measured based on the oxidation of NADH (Equation (2)) whilst the enzyme redox reaction consumed NH_4_^+^ ion in the process. Thus, the redox current is proportional to the NH_4_^+^ ion concentration changes under optimal conditions at an applied potential of +0.55 V:

(1) $$\begin{array}{c} \text{AlaDH}\\ \text{Pyruvate}+\text{NADH}+{\text{NH}}_{4}^{+}\to \text{L-alanine}+{\text{NAD}}^{+}+H_{2}O \end{array}$$

(2) $$\text{NADH}\to {\text{NAD}}^{+}+H^{+}+{2e}^{-}$$

**Figure 1.**
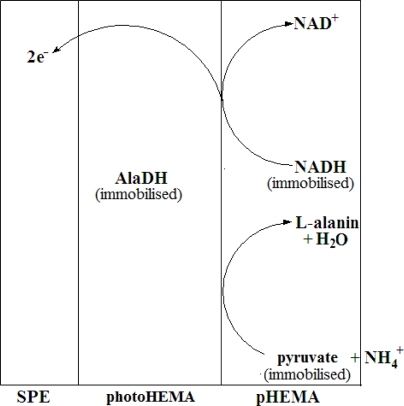
The mechanism of enzymatic reaction involved in the NH_4_^+^ biosensor based on stacked membranes.

To construct the stacked membrane biosensor, AlaDH enzyme was first entrapped in the photoHEMA membrane, whereby the membrane with the entrapped enzyme was formed via UV photopolymerisation of 2-hydroxylethyl methacrylate monomer. Past studies have shown that the use of photoHEMA is compatible with many enzymes without leaching problems. To further immobilize the pyruvate and NADH, a low molecular weight poly(2-hydroxylethyl methacrylate) (pHEMA) membrane containing both of these substances was cast on top of the enzyme membrane after photocuring. The use of this second membrane allowed both pyruvate and NADH to be immobilized but their free diffusion in the membrane would also be ensured to maintain a rapid reaction rate. The biosensor was thus designed to provide a reagentless system specific for NH_4_^+^ ion. Figure 1 represents the mechanism of the enzymic reaction involved in the developed NH_4_^+^ ion biosensor based on a stacked membrane system.

## 2. Experimental

### 2.1. Reagents

Chemicals were obtained from commercial sources and were used without further purification. β-Nicotinamide adenine dinucleotide, reduced form (NADH, 98%) was purchased from Sigma. Stock solution of L-alanine dehydrogenase enzyme (AlaDH, E.C. 1.4.1.1, from *Bacillus subtillis*, Sigma) was prepared by mixing an appropriate amount of AlaDH enzyme solution with 10 mM phosphate buffer pH 7 in an Eppendorf tube and stored at 4 °C [23]. Sodium pyruvate (C_3_H_3_NaO_3_, 99%, Sigma) stock solution was prepared by dissolving an appropriate amount of pyruvate salt in deionised water. pH 7, 10 mM phosphate buffer was prepared by adding 10 mM dipotassium hydrogen phosphate (K_2_HPO_4_, 98%, Fluka) to 10 mM potassium dihydrogen phosphate (KH_2_PO_4_, 99.5%, Fluka) and adjusting to the required pH value [23]. A standard stock ammonia solution was prepared by dissolving the required amount of concentrated ammonia solution (NH_4_OH, 25%, Merck) in deionised water. The ammonia solution used was standardised by the Nessler method using ammonium chloride (NH_4_Cl, 99.5%) salt. A homogeneous stock solution of 2-hydroxyethyl methacrylate monomer (HEMA, C(CH_3_)COOCH_2_CH_2_OH, 97%, Aldrich) was prepared by mixing appropriate amounts of HEMA monomer and initiator (2,2-dimethoxy-2-phenylacetophenone, DMPP, C_16_H_16_O_3_, 98%, Fluka) in a vial wrapped with aluminium foil. The mixture was then stirred gently for a few minutes and stored at 4 °C. Poly(2-hydroxyethyl methacrylate) (pHEMA) was obtained commercially from Aldrich.

### 2.2. Instrumentation

Electrochemical behaviours of the reagentless biosensor were characterised using a chronoamperometry technique with an Autolab PG12 (AUT 71681) Potentiostat/Galvanostat. A conventional three-electrode electrochemical cell was used with a glassy carbon electrode as an auxiliary electrode. A Ag/AgCl electrode saturated with KCl was used as a reference and a modified carbon paste electrode was used as a working electrode. The SPEs used were designed by Universiti Kebangsaan Malaysia and manufactured by Scrint Print Co. All potentials were measured and reported *versus* a Ag/AgCl electrode (saturated by KCl). During the constant potential experiments, a magnetic stir bar was used and the background current was allowed to decay to a constant value before NH_4_^+^ ion was added to the buffer solution. Measurements of pH were made with a pH-meter (MeterLab PHM 210). AlaDH enzyme-containing photoHEMA membrane was prepared by UV-initiated photopolymerisation with an UV-exposure unit (RS Components 196-5251).

### 2.3. Construction of Biosensor

Reagentless NH_4_^+^ biosensor was constructed by depositing 3 μL of 2.98 mg AlaDH/g of HEMA monomer mixture onto the SPE and exposing it to long-wave ultraviolet radiation for 500 s with extensive nitrogen gas purging. Next, appropriate amounts of NADH and pyruvate were dissolved into an appropriate amount of pHEMA solution prepared by dissolving 50 mg of the polymer in 20% 1,4-dioxane in water. This was then deposited on the photocured membrane containing AlaDH enzyme and left to dry at 4 °C for 24 h to form the second membrane layer.

### 2.4. Optimisation of Biosensor Responses

All electrochemical experiments were performed at room temperature in an undivided three-electrode cell containing supporting electrolyte solution (4 mL of 10 mM phosphate buffer pH 7) under constant stirring conditions (100 rpm). NH_4_^+^ ion was injected at the electrode surface in the measurement cell after stabilisation of the baseline current. The measurements taken were expressed as the current difference in the absence and presence of NH_4_^+^ ion.

Biosensor response was studied using the cyclic voltammetry technique between −1.00–1.00 V *versus* Ag/AgCl at a scan rate of 0.02 V/s. The dependence of the amperometric signal on the applied potential was examined in the range of 0.45–0.65 V *versus* Ag/AgCl. The pH effect was studied by varying the pH of the electrolyte solution in the range of pH 5.8–8.0 using 10 mM potassium phosphate buffer. For the optimisation of enzyme loading in the photoHEMA membrane, enzyme membranes were prepared from 5 μL mixtures of AlaDH and monomer HEMA (1:1) in different enzyme loadings (0.44–4.10 mgAlaDH/g photoHEMA).

Enzyme electrodes with different membrane thicknesses were prepared by changing the mixture volumes of monomer HEMA and AlaDH (1:1) in the range of 2–6 μL while the enzyme loading was kept constant at 2.98 mg/g photoHEMA. The thicknesses of the membranes were measured using a digital Vernier caliper after exposure of the sensing membranes to UV radiation.

Biosensor response to temperature changes in the 10–50 °C range was determined by varing the temperature of the electrolyte solution in a thermostatic bath. For this study, about 21 biosensors were prepared a day before and stored overnight at 4 °C before testing was performed.

For the optimisation of the respective NADH and pyruvate loadings in the pHEMA layer, various loadings of pyruvate (3.74–163.11 mg/g pHEMA) and NADH (22.98–1,149.11 mg/g pHEMA) were used. After optimisation of the experimental conditions, the effect on the reagentless biosensor’s performance was investigated against different NH_4_^+^ ion concentrations in the range of 10–600 mM.

The reproducibility of the biosensor was assessed using different biosensors and each biosensor was tested once by using 30 mM NH_4_^+^ ion, whereas the repeatability of the biosensor was examined using the same biosensor tested in a concentration of 30 mM NH_4_^+^ ion and five consecutive measurements were taken for every biosensor.

The shelf life of the biosensor was examined by determining 30 mM NH_4_^+^ ion over a period of 30 days. All of the electrodes were tested once on the first day and kept at 4 °C while not in use. After that, each of these biosensors was tested twice only according to a specified date.

Interferences with the NH_4_^+^ ion biosensor were investigated by using interferents such as Na^+^, K^+^, methylamine (CH_3_NH_2_) and ethylamine (C_2_H_5_NH_2_). The molar ratio of interferents to NH_4_^+^ ion was varied in the range of 0.01–10. The degree of interference of these interferents was evaluated separately with amperometric measurements recorded in the presence and absence of the interferent in the determination of 30 mM NH_4_^+^ ion. Statistical *t*-tests were employed to compare the responses obtained in the absence and presence of interferent.

Analysis of water samples was performed using five real water samples collected at different points along a river. Water samples presenting suspended particles were filtered through a 0.45 μm cellulose acetate membrane filter (Whatman) and the pH was adjusted to pH 7 using 10 mM K_2_HPO_4_ and KH_2_PO_4_. Recovery tests were conducted by adding standard concentrations of NH_4_^+^ ion in the linear range to the water samples. The results were then validated using established procedure of Nesslers’ method.

## 3. Results and Discussion

### 3.1. The Electrochemistry of Immobilized AlaDH

**Figure 2.**
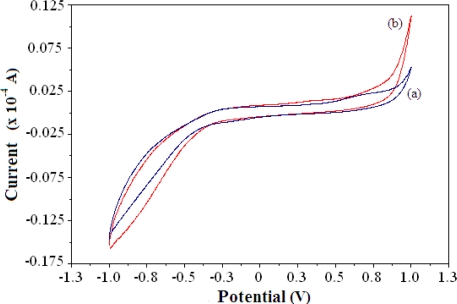
Cyclic voltammograms of NH_4_^+^ ion biosensor in the absence (**a**) and presence (**b**) of 100 mM NH_4_^+^ ion at pH 7 with a scan rate of 0.02 V/s *versus* Ag/AgCl electrode.

Due to the enzymatic reaction of AlaDH and the pyruvate substrate, the signal from the electrocatalytic oxidation of NADH was proportional to the NH_4_^+^ ion concentration. Figure 2 shows a typical cyclic voltammogram of the reagentless NH_4_^+^ ion biosensor demonstrating the electrocatalytic activity of biosensor in the absence and presence of 100 mM NH_4_^+^ ion. The oxidation current of the biosensor increases after NH_4_^+^ ion addition in the positive potential region. Although no clearly defined cyclic voltammetric peak appears, the biosensor shows an increase in current in response to NH_4_^+^ ion in the potential range of 0.4–0.9, as indicated by the cyclic voltammogram.

The increase of the current was attributed to the electrocatalytic oxidation of NADH during enzymatic conversion of pyruvate to L-alanine in the presence of NH_4_^+^ ion. The electrode reaction at the electrode surface is governed by a single one-electron step in an overall two-electron process, where NADH is irreversibly oxidized through loss of an electron to produce a cation radical NADH•^+^ and is expressed by Equation (3):

(3) $$\text{NADH}\to {\text{NADH}•}^{+}+e^{-}$$

which then deprotonates to produce a neutral radical NAD• as written below:

(4) $${\text{NADH}•}^{+}\to \text{NAD}•+H^{+}$$

NAD• is immediately oxidized to NAD^+^ at the electrode surface at the positive potential involved as described by Equation (5):

(5) $$\text{NAD}•\to {\text{NAD}}^{+}+e^{-}$$

**Figure 3.**
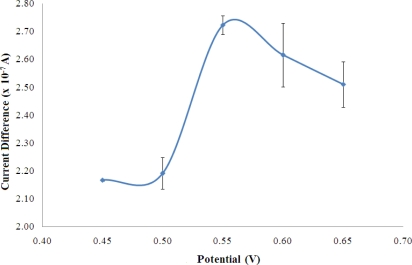
The dependence of the bisoensor response on the applied electrode potential in a solution containing 100 mM NH_4_^+^ ion at pH 7 (n = 3).

The net electrochemical oxidation of NADH could be described by Equation (2) [24]:

Figure 3 shows the biosensor response of a fixed amount of NH_4_^+^ ion when the potential was varied between 0.45–0.65 V and optimum applied potential was observed at +0.55 V, where the highest current was obtained. The working potential was found like the NADH oxidation potential using a 4,6-diamino-2-mercaptopyrimidine modified gold (Au) electrode [25] and a thiocytosine modified Au electrode [26]. However, the oxidation of NADH at +0.55 V was found lower compared to the electrochemical oxidation of NADH employing boron-doped diamond electrode (+0.58 V) [27]. The potential of +0.55 V was used in this work for the amperometric detection of NH_4_^+^ ion.

### 3.2. Optimization Studies on Biosensor Operation

#### 3.2.1. Optimisation of pH

**Figure 4.**
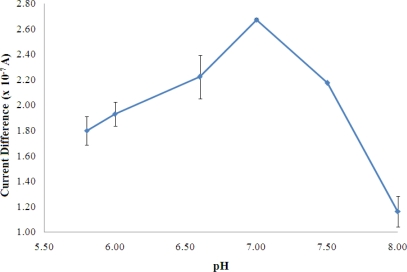
The effect of pH on the biosensor response in a solution containing 100 mM NH_4_^+^ ion (n = 3).

For the optimisation of buffer pH, as observed in Figure 4, the maximum enzyme reaction occurred at pH 7 and it is the optimum pH for reversible behavior of AlaDH as reported in the literature [28].

The rate of an enzyme reaction, in most cases, passes through a maximum as a function of pH. From Figure 3, at pH above 7, the enzyme may undergo an irreversible denaturation where the activity may not be restored even after readjustment of the pH to the optimal value [29].

#### 3.2.2. Optimisation of Enzyme Loading for Biosensor

**Figure 5.**
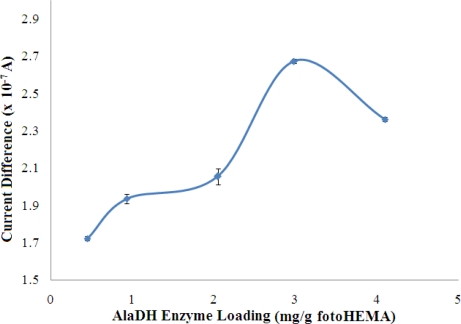
Effect of enzyme loading on biosensor response in a solution containing 100 mM NH_4_^+^ ion at pH 7 (n = 3).

The effect of enzyme loading on the biosensor response is shown in Figure 5. The response increased with increasing AlaDH enzyme loading in the membrane until saturation was achieved at an enzyme loading of 2.98 mg AlaDH/g fotoHEMA. However, the response was lower after this optimal loading.

Typically, the rate of the enzymatic reaction is directly proportional to the enzyme loading. There are several conditions under which this proportionality may not occur. When large amounts of enzyme are used the added cofactor NADH may bind to the enzyme and not be available for reaction [29]. High enzyme loading also results in the blocking of the enzyme active sites, especially for enzyme that is located far from the surface of the membrane, thus making some of the enzyme not participate in enzymatic reactions. Furthermore, excessive loading of enzyme will also create a diffusion barrier to the movement of substrates and reaction products, which will also reduce the response obtained. Thus, the optimised enzyme loading of 2.98 mg/g photoHEMA was used throughout the experiments. Kwan *et al.* [30] have reported using the same enzyme entrapped by a poly(carbamoyl) sulfonate hydrogel on a Teflon membrane for amperometric determination of alanine. The backward enzyme reaction involved specific dehydrogenation of alanine that consumed NAD^+^. The reaction products, pyruvate and NADH were used to initiate the two other reactions that follow, which involved salicylate hydroxylase and pyruvate oxidase [30].

#### 3.2.3. Optimisation of Membrane Thickness for Biosensor Fabrication

**Figure 6.**
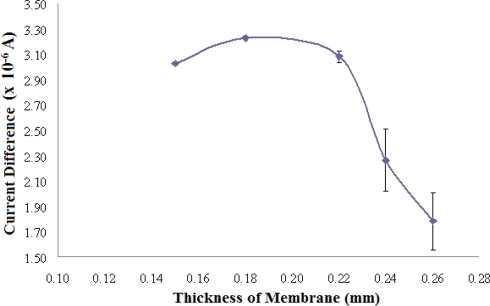
Effect of membrane thickness on the biosensor response in a solution containing 100 mM NH_4_^+^ ion at pH 7 (n = 3).

The effect of the thickness of photoHEMA membranes containing 2.98 mg AlaDH/g photo HEMA on the biosensor response is presented in Figure 6. The optimum thickness of AlaDH enzyme-containing photoHEMA membrane was found to be 0.18 mm because of the consistent high signal attained for the same NH_4_^+^ ion concentration (RSD = 5.6%). A membrane thickness of 0.15 mm gave a slightly lower response due to the low reproducibility of preparing a thin membrane (RSD = 14.2%). Moreover, a thin membrane may lead to the leaching out of the immobilized enzyme. Thicker membranes, between 0.22–0.26 mm, gave inconsistently low responses as these membranes resulted in the active sites of the enzyme becoming less accessible, in addition to hindering the diffusion of rectants and products.

#### 3.2.4. Optimisation of Temperature of Biosensor Operation

**Figure 7.**
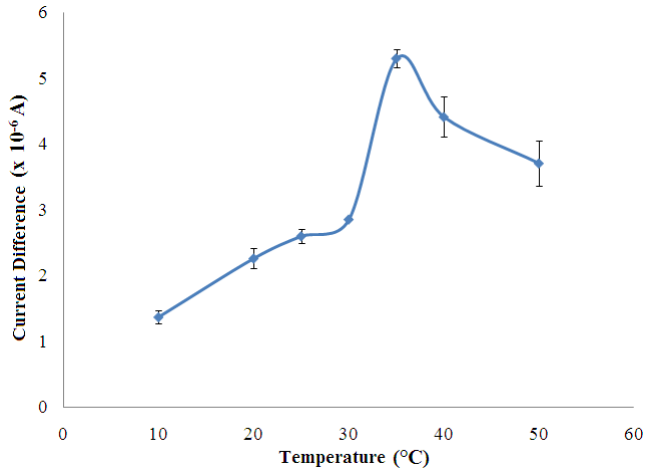
The temperature effect on the response of the NH_4_^+^ ion biosensor in a solution containing 100 mM NH_4_^+^ ion at pH 7 (n = 3). The biosensors were prepared one day before and stored at 4 °C before testing at various temperatures.

The biosensor response increased rapidly with temperature and reached a maximum at the temperature of 35 °C (Figure 7). This is explained by the increase in the rate of an enzyme reaction with increasing temperature [29].

However, the biosensor response decreased when the temperature increased above 35 °C. In this state, the denaturation of the enzyme occurs where the weak bonding such as hydrogen and ionic bonds are broken at high temperatures [31]. In the present work, 25 °C was chosen as working temperature for all subsequent experiments since the sensitivity of the biosensors were sufficiently good for further studies.

#### 3.2.5. Optimisation of Loading for Pyruvate and NADH

**Figure 8.**
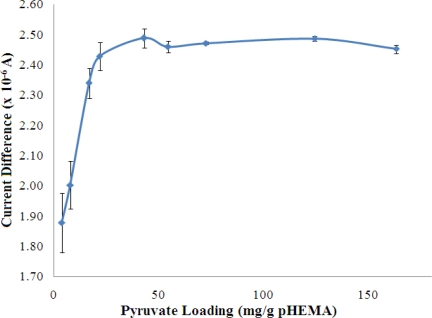
The effect of the amount of pyruvate entrapped in a pHEMA membrane on the response of the NH_4_^+^ ion biosensor (100 mM NH_4_^+^ ion at pH 7) (n = 3).

**Figure 9.**
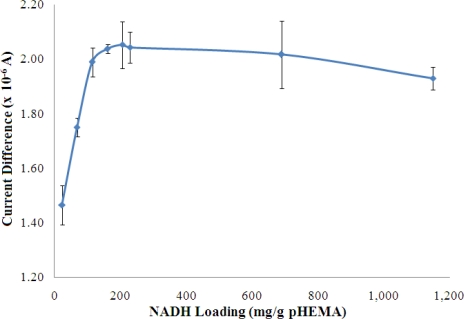
The response of the NH_4_^+^ ion biosensor to various amount of NADH entrapped in a pHEMA membrane on the response of the NH_4_^+^ ion biosensor (100 mM NH_4_^+^ ion at pH 7) (n = 3).

The concentrations of both pyruvate and NADH, which were immobilized in the pHEMA membrane were optimized to yield the best biosensor response to NH_4_^+^ ion. The results are shown in Figures 8 and 9, which indicate that the current response increases with increasing pyruvate and NADH loadings and becomes saturated at loadings of 42.99 mg pyruvate/g pHEMA and 160.87 mg NADH/g pHEMA, respectively.

### 3.3. Analytical Performance of NH_4_^+^ Ion Biosensor

#### 3.3.1. The Response Range of Biosensor

**Figure 10.**
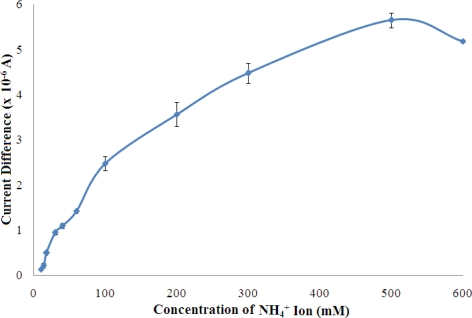
The response of the biosensor towards different NH_4_^+^ ion concentration in the range of 10–600 mM at pH 7 (n = 3).

The response of the biosensor was examined from 10–600 mM of NH_4_^+^ ion and the results are shown in Figure 10.

**Figure 11.**
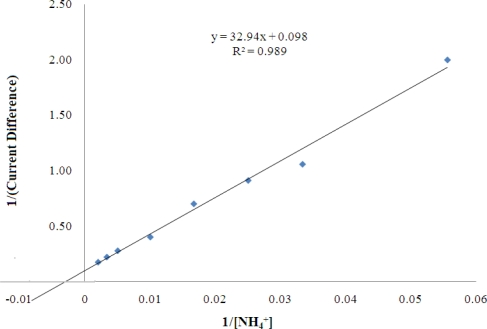
Lineweaver-Burk plots of the reagentless biosensor.

The biosensor sensor response was found increased with increasing NH_4_^+^ ion concentration from 10–500 mM, and reached saturation beyond 500 mM NH_4_^+^ ion. This phenomenon is due to the fact that available pyruvate, cofactor NADH, and enzyme active sites have been gradually consumed with increasing NH_4_^+^ ion concentration until a saturation condition is achieved. Furthermore, at high levels of NH_4_^+^ ion, the ionic strength of the reaction medium increases and thereby reduces the enzymatic reaction which is also influenced by the charge at the enzyme active sites. A linear response range was observed at 10–100 mM (R^2^ = 0.978, n = 7) with a limit of detection of 0.18 mM. The apparent *K*_m_ value estimated from the Lineweaver-Burk plot was 34.01 mM (Figure 11).

#### 3.3.2. The Biosensor Reproducibility and Repeatability

**Figure 12.**
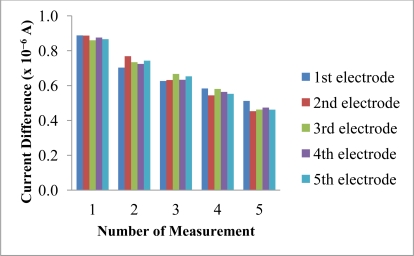
The reproducibility and repeatability of the reagentless NH_4_^+^ ion biosensor that has been exposed to 30 mM NH_4_^+^ ion at pH 7 (n = 5).

The reproducibility and repeatability of the reagentless biosensor was evaluated at 30 mM NH_4_^+^ ion. The current difference measurements obtained using different electrodes gave low RSD values from 1.4–4.9% (n = 5). These values were low enough to consider that the membranes were reproducible as fabricated by the proposed method. However, the slightly higher repeatability RSD values (21.7–26.4%, n = 5) obtained using the same biosensor was attributed to the irreversible oxidation of immobilized NADH at the electrode surface, which resulted in a decreasing current difference being observed for five consecutive measurements over the same electrode (Figure 12). The poor repeatability is an inherent effect from the leaching of the immobilized NADH and pyruvate. Both of these reactants are deliberately allowed to leach out from the membrane in order for them to diffuse into the enzymatic membrane to react with the AlaDH enzyme. Therefore, the poor repeatability is attributed to the loss and consumption of these reactants when the biosensor is reused many times. The biosensor is mainly used for on-site analysis and because it is made from SPE that can be manufactured at low cost, thus one can aim for a single-use and disposable electrode.

#### 3.3.3. Biosensor Stability

In terms of biosensor stability, over a period of 30 days and using 30 mM NH_4_^+^ ion, the reagentless biosensor first showed a decrease in sensitivity of approximately 67% during the first four days, after which the sensitivity was maintained for up to 20 days before further deterioration of the response began. After 30-day storage period, the biosensor still retained about 50% of the initial sensitivity. (Table 1). An amperometric biosensor employing AlaDH enzyme for alanine determination reported an operational stability of only two days and the sensor response dropped to 50% of the initial sensitivity after day-13 [30].

#### 3.3.4. Effects of Interferents on Biosensor Response

The possible interferences in the determination of NH_4_^+^ ion by the reagentless biosensor were investigated and the results are depicted in Table 2. The reagentless biosensor did not suffer any interference from alkali metal ions at levels occurring in natural waters [32]. But approaching a 1:1 molar ratio, interference was observed because at high concentration of ions, and high ionic strength of the measurement medium that appears to reduce the enzymatic reaction [31]. The stability of AlaDH is higher in the presence of NaCl salt compared to KCl [22], hence Na^+^ ion did not demonstrate any observable interference when compared with K^+^ ion, even at a molar ratio of 1:1. CH_3_NH_2_ and C_2_H_5_NH_2_ interfered significantly at molar ratios of 0.1 and above. The interference increases the biosensor response as some physicochemical properties of amines are similar to NH_4_^+^ ion. However, such interference by amines is not expected in natural water samples due to their low level, usually 100 times lower than NH_4_^+^ ion [10].

#### 3.3.5. Performance of Biosensor for Water Sample Analysis

To assess the accuracy of the biosensor, recovery studies were performed using river water samples which had been spiked with known NH_4_^+^ ion concentrations within the linear response range of the biosensor. The results are shown in Table 3. The recovery values obtained were close to 100% in the five different NH_4_^+^ ion concentrations spiked into river water samples. The analytical performance of the reagentless biosensor for water analysis was compared with the standard method of Nessler method using spectrophotometric method. The *t*-test applied to examine whether the two methods gave results that both methods were not statistically different for NH_4_^+^ ion determination. This indicates that the reagentless biosensor can be used for NH_4_^+^ ion determination.

#### 3.3.6. Comparison with Similar Biosensors

Amperometric based enzymic biosensors for NH_4_^+^ ion determination using GLDH enzyme are commonly reported in the literature [17–19] (Table 4), however, they all require that the substrates involved in the enzymic reaction with GLDH enzyme, be introduced into the assay solutions. The linear detection ranges of NH_4_^+^ ion for biosensors using GLDH were somehow narrower compared to the use of AlaDH enzyme. The GLDH enzyme system has also been used for the optical detection of NH_4_^+^ ion in water samples at μM level based on NADH oxidation [33]. A chitosan matrix was used for the immobilization of GLDH enzyme on a supporting glass slide but this optical biosensor system for NH_4_^+^ ion determination suffered interference from other possible cations present in the water samples.

## 4. Conclusions

A new reagentless amperometric biosensor for the determination of NH_4_^+^ ions based on the enzyme AlaDH and involving the use of a stacked membrane system for the immobilization of all reagents was successfully developed. The use of a layer of low molecular weight pHEMA for NADH and pyruvate immobilization and a photocured photoHEMA membrane with entrapped AlaDH enzyme offers a simple and rapid biosensor fabrication procedure in addition to convenient NH_4_^+^ ion analysis, particularly for performing on-site analysis without the need of any addition of reagents. The reagentless biosensor demonstrated a wide linear response range between 10–100 mM NH_4_^+^ ion with a detection limit of 0.18 mM and good selectivity over some major cations. Such a biosensor has a potential to overcome the problem of on-site analysis of NH_4_^+^ ion because it can be used for sewage NH_4_^+^ ion analysis without further dilution (NH_4_^+^ ion concentrations of sewage are typically from 65–170 mM).

## Figures and Tables

**Table 1.** The stability of the reagentless NH_4_^+^ ion biosensor tested in solution containing 30 mM NH_4_^+^ ion at pH 7 over a period of 30 days (n = 3).

**Time (day)**	**Current Difference (×10^−7^ A)**	**Standard deviation**	**Relative response (%)**
1	0.9541	0.0695	100.0
4	0.6354	0.0555	66.6
19	0.5754	0.0062	60.3
23	0.5099	0.0418	53.4
26	0.4697	0.0059	49.2
30	0.4490	0.0378	47.1

**Table 2.** The effect of Na^+^, K^+^, CH_3_NH_2_ and C_2_H_5_NH_2_ at different molar ratios to the response of NH_4_^+^ ion biosensor (fixed NH_4_^+^ at 30 mM; pH 7; n = 3).

**Interferent**	**Current difference (×10^−6^ A) at different molar ratios of interferent: NH_4_^+^ ion**				
	**0.00:1**	**0.01:1**	**0.10:1**	**0.50:1**	**1.00:1**
Na^+^	0.887 ± 0.013	0.850 ± 0.033	0.843 ± 0.016	0.831 ± 0.036	0.844 ± 0.039
K^+^	0.887 ± 0.013	0.863 ± 0.113	0.866 ± 0.086	0.901 ± 0.049	0.797 ± 0.036 *
CH_3_NH_2_	0.887 ± 0.013	0.877 ± 0.033	1.013 ± 0.001 *	1.005 ± 0.007 *	1.778 ± 0.061 *
C_2_H_5_NH_2_	0.887 ± 0.013	0.851 ± 0.042	0.825 ± 0.024 *	1.114 ± 0.017 *	1.599 ± 0.110 *

* *t* value > *t* critical value at 95% confident level with 4 degrees of freedom.

**Table 3.** A comparative study of the reagentless biosensor and Nessler method for NH_4_^+^ ion analysis in different river water samples containing spiked NH_4_^+^ ion concentrations from 10–30 mM.

**Sample**	**Concentration of NH_4_^+^ ion spiked in each sample (mM)**	**Concentration of NH_4_^+^ ion determined by biosensor (n = 3), (mM)**	**Concentration of NH_4_^+^ ion by Nessler method (n = 3), mM**	**Recovery (%)**
Sample 1	10.00	10.29 ± 0.95	10.41 ± 0.79	102.9
Sample 2	18.00	18.63 ± 1.05	18.20 ± 0.91	103.5
Sample 3	22.00	21.56 ± 1.52	21.90 ± 0.76	98.0
Sample 4	26.00	26.13 ± 0.32	26.10 ± 0.15	100.5
Sample 5	30.00	29.01 ± 2.33	30.38 ± 1.20	96.7

**Table 4.** The comparison between reagentless NH_4_^+^ biosensor and reported amperometric NH_4_^+^ biosensor.

**Parameters**	**Present study**	**Abass *et al.* [17]**	**Bertocchi *et al.* [18]**	**Kwan *et al.* [19]**
Enzyme	AlaDH	GLDH	GLDH	GLDH & glutamate oxidase
Dynamic linear range (mM)	10–100	0.0017–0.25	0.01–0.3	0.01–0.30
Detection limit (mM)	0.18	0.0017	0.01	0.002
Response time (min)	<3	–	–	4
